# Phenol-Soluble Modulin Toxins of *Staphylococcus haemolyticus*

**DOI:** 10.3389/fcimb.2017.00206

**Published:** 2017-05-24

**Authors:** Fei Da, Hwang-Soo Joo, Gordon Y. C. Cheung, Amer E. Villaruz, Holger Rohde, Xiaoxing Luo, Michael Otto

**Affiliations:** ^1^Pathogen Molecular Genetics Section, Laboratory of Bacteriology, National Institute of Allergy and Infectious Diseases, National Institutes of HealthBethesda, MD, United States; ^2^Department of Pharmacology, School of Pharmacy, Fourth Military Medical UniversityXi'an, China; ^3^Institute of Medical Microbiology, Virology, and Hygiene, University Hospital Hamburg-EppendorfHamburg, Germany

**Keywords:** *Staphylococcus haemolyticus*, coagulase-negative staphylococci, phenol-soluble modulin, toxin, hemolysin, leukocidin

## Abstract

Coagulase-negative staphylococci (CoNS) are important nosocomial pathogens and the leading cause of sepsis. The second most frequently implicated species, after *Staphylococcus epidermidis*, is *Staphylococcus haemolyticus*. However, we have a significant lack of knowledge about what causes virulence of *S. haemolyticus*, as virulence factors of this pathogen have remained virtually unexplored. In contrast to the aggressive pathogen *Staphylococcus aureus*, toxin production has traditionally not been associated with CoNS. Recent findings have suggested that phenol-soluble modulins (PSMs), amphipathic peptide toxins with broad cytolytic activity, are widespread in staphylococci, but there has been no systematic assessment of PSM production in CoNS other than *S. epidermidis*. Here, we identified, purified, and characterized PSMs of *S. haemolyticus*. We found three PSMs of the β-type, which correspond to peptides that before were described to have anti-gonococcal activity. We also detected an α-type PSM that has not previously been described. Furthermore, we confirmed that *S. haemolyticus* does not produce a δ-toxin, as results from genome sequencing had indicated. All four *S. haemolyticus* PSMs had strong pro-inflammatory activity, promoting neutrophil chemotaxis. Notably, we identified in particular the novel α-type PSM, *S. haemolyticus* PSMα, as a potent hemolysin and leukocidin. For the first time, our study describes toxins of this important staphylococcal pathogen with the potential to have a significant impact on virulence during blood infection and sepsis.

## Introduction

Sepsis is a severe infection of the blood and the most common cause of death in hospitalized patients (Dellinger et al., 2008). It is estimated that about one million people develop sepsis every year in the U.S., and in 28–50% this is fatal (Jawad et al., 2012). Coagulase-negative staphylococci (CoNS) are a leading cause of sepsis, and the most frequent cause of sepsis in neonates (Cheung and Otto, 2010). *Staphylococcus haemolyticus* is considered the most frequent etiological agent causing CoNS infections after *S. epidermidis* (Czekaj et al., 2015) and the by far most important non-*S. epidermidis* CoNS species involved with blood infections (Becker et al., 2014; Hitzenbichler et al., 2016).

The overwhelming immune response that is characteristic for sepsis and the reason for its symptoms, such as fever, chills, tachycardia, and tachypnea, has commonly been believed to be due to pro-inflammatory, invariant structures on the bacterial surface, such as lipoteichoic acid in Gram-positive bacteria (Fournier and Philpott, 2005). In addition to those conserved surface structures, superantigenic toxins possibly trigger sepsis (Holtfreter and Broker, 2005), but superantigenic toxins only occur in *S. aureus* but not CoNS. Recently, we have provided proof-of-principle that also toxins that occur in CoNS, namely the phenol-soluble modulin (PSM) toxin family, promote sepsis (Qin et al., 2017). This was shown for the mobile-genetic element-encoded PSM-mec, which occurs in a series of methicillin-resistant CoNS as well as *S. aureus* (Queck et al., 2009). Direct evidence showing a similar role for the other, commonly core genome-encoded members of the PSM family (Peschel and Otto, 2013; Cheung et al., 2014a) is still lacking, which is mainly due to the fact that there are multiple PSM-encoding genetic loci and the construction of isogenic, multiple deletion mutants in CoNS is a difficult task. However, based on the conserved structure of PSMs and the fact that all PSM types have been shown to activate immune cells via recognition by the formyl peptide receptor 2 (FPR2) (Kretschmer et al., 2010), it is fair to assume that other PSMs may also promote sepsis.

While mass-spectrometric analysis of CoNS culture filtrates indicated that members of the PSM family occur in most CoNS species (Rautenberg et al., 2011), the identification of PSMs is difficult and cannot be based on genome comparisons alone. This is because (i) PSMs considerably vary in DNA and amino acid sequence between species (Cheung et al., 2014a), and (ii) PSM-encoding genes can be very short—the smallest PSMs identified to date are 20 amino acids in length (Cheung et al., 2014a). PSMs are subdivided into two groups; the somewhat longer β-type PSMs (~45 amino acids) and the shorter α-type PSMs (~20–25 amino acids), to which also the long-known δ-toxin belongs. Furthermore, PSMs do not have a signal peptide and are secreted as the primary, N-formylated translation product by dedicated ABC exporter systems (Chatterjee et al., 2013; Yoshikai et al., 2016). For these reasons, identification of PSMs requires analysis of culture filtrates and often, peptide purification, mass-spectrometry, and/or N-terminal sequencing to determine the nature of PSMs in a given species. Such a systematic analysis of the PSM composition has only been performed in *S. aureus* and *S. epidermidis*, but not in other important pathogenic staphylococcal species, such as *S. haemolyticus*.

All PSMs share as a characteristic feature the formation of amphipathic α-helices, giving them surfactant properties (Cheung et al., 2014a). In the α-type PSMs, this amphipathic α-helix stretches over virtually the entire peptide, while *in-silico* analysis indicated that β-type PSMs have such a helix at the C-terminal part (Cheung et al., 2014a) and structural investigation by nuclear magnetic resonance spectroscopy has shown recently that also the N-terminus in β-type PSMs forms an α-helix (Towle et al., 2016). Studies in *S. epidermidis* and *S. aureus* have shown that PSMs have multiple functions in staphylococcal physiology and pathogenesis (Peschel and Otto, 2013; Cheung et al., 2014a). In addition to the FPR2-receptor mediated pro-inflammatory function that leads to neutrophil chemotaxis, cytokine release, and other inflammatory phenotypes (Wang et al., 2007; Cheung et al., 2010; Kretschmer et al., 2010), they are broadly cytolytic, killing many cell types including leukocytes and erythrocytes, in a presumably receptor-independent fashion by membrane destruction (Wang et al., 2007; Cheung et al., 2010). Furthermore, they structure biofilms and lead to biofilm dispersal (Wang et al., 2011; Periasamy et al., 2012). In *S. aureus*, the investigation of isogenic *psm*-negative mutants has shown a particularly strong impact of particularly the *S. aureus* PSMα peptides on blood and skin infection.

In addition to the preliminary analysis of *S. haemolyticus* culture filtrate by high-performance reversed-phase chromatograph/mass spectrometry (HPLC/MS) that we performed recently (Rautenberg et al., 2011), there is some previous evidence in the literature suggesting that *S. haemolyticus* produces PSM peptides. Namely, Watson et al. characterized three substances previously found to have anti-gonococcal activity (Frenette et al., 1984) as peptides with lengths and amino acid sequences that according to contemporary classification would be regarded as β-type PSMs (Watson et al., 1988). Later investigation indicated that the anti-gonococcal activity of those peptides is due to membrane destruction (Frenette et al., 1988). However, these peptides were never analyzed for the more important features of PSMs in human pathogenesis, such as their pro-inflammatory and cytolytic activities toward human cells. Most notably, they were never implicated in the hemolytic activity that gave the species its name. While the hemolytic activity of *S. haemolyticus* has been attributed to δ-toxin-like peptides, these were never further characterized as for their specific size and amino acid sequence (Loyer et al., 1990).

In the present study, we performed a systematic analysis of PSMs of *S. haemolyticus*. We found that *S. haemolyticus* produces the three previously described anti-gonococcal growth inhibitors, which we now describe as *S. haemolyticus* PSMβ peptides. Importantly, we found that *S. haemolyticus* produces a not previously described α-type PSM with pronounced cytolytic capacity significantly surpassing cytolytic capacities of the *S. haemolyticus* PSMβ peptides. Our findings indicate that the virulence of *S. haemolyticus*, including hemolytic capacity as its most notable feature, is due to members of the PSM family.

## Materials and methods

### Ethics statement

This study was carried out in accordance with the recommendations of approved protocols at the NIH Blood Bank or with a protocol (633/2012BO2) approved by the Institutional Review Board for Human Subjects, NIAID, NIH with written informed consent from all subjects. All subjects gave written informed consent in accordance with the Declaration of Helsinki. *S. haemolyticus* isolates were collected in an anonymous fashion, stored and analyzed according to German law and standards for research use of human biological material.

### Bacterial strains and growth conditions

Bacteria were grown in tryptic soy broth (TSB) at 37°C with shaking. The *S. haemolyticus* strain ATCC29970 was used in all experiments. Clinical *S. haemolyticus* isolates were grown from blood cultures obtained from patients hospitalized in the University Medical Center Hamburg-Eppendorf. Species identification was performed by MALDI-TOF mass spectrometry fingerprinting using a MALDI-TOF Biotyper instrument (Bruker Daltonics, Bremen, Germany). All isolates were stored at −80°C for subsequent characterization.

### Peptides

PSM peptides were synthesized by commercial vendors at >95% purity (*S. aureus* PSMα3, American Peptide Company; *S. haemolyticus* PSMα, Peptide 2.0; *S. haemolyticus* PSMβ1, β2, and β3, Atlantic Peptide) with the N-terminal N-formyl methionine modification that is introduced to all bacterial proteins and remains in PSMs due to the lack of a signal peptide.

### Analysis of PSMs by RP-HPLC/MS

PSMs were analyzed by a reversed-phase high performance chromatography/electrospray ionization mass spectrometry (RP-HPLC/ESI-MS) method using an Agilent 1260 Infinity chromatography system coupled to a 6120 Quadrupole LC/MS in principle as described (Joo and Otto, 2014), but with a shorter column and a method that was adjusted accordingly. A 2.1 × 5 mm Brownlee SPP (2.7 μm) guard column was used at a flow rate of 0.5 ml/min. After sample injection, the column was washed for 0.5 min with 90% buffer A [0.1% trifluoroacetic acid (TFA) in water]/10% buffer B (0.1% TFA in acetonitrile), then for 3 min with 25% buffer B. Then, an elution gradient was applied from 25 to 100% buffer B in 2.5 min, after which the column was subjected to 2.5 min of 100% buffer B to finalize elution.

### PSM purification

To purify *S. haemolyticus* PSMs, two-step reversed phase chromatography on an AKTA 100 purifier system (GE Healthcare) was used. Forty milliliters of a stationary-phase culture of *S. haemolyticus* was injected on an HR 16/20 column packed with SOURCE PHE (GE Healthcare) material (~16 ml bed volume). The flow rate was 4 ml/min. After injection, the column was washed with 3 column volumes of buffer A (0.1% TFA in 10% acetonitrile/90% water). Afterwards, bound material was eluted with a linear gradient over 15 column volumes from 100% buffer A to 100% buffer B (0.1% TFA in 90% acetonitrile/10% water). Fractions in the elution range of PSMs were pooled, lyophilized, and re-dissolved in 2 ml water.

Pooled and re-dissolved PSM fractions were injected onto a ZORBAX SB-C18 9.4 × 25 cm (Agilent) column, which was run at 4 ml/min. A linear gradient was applied over 80 min from 100% buffer A to 100% buffer B. Peak fractionation was used and PSM-containing peaks were further analyzed for containing peptides and their purity by RP-HPLC/MS.

### N-terminal sequencing

PSMα of *S. haemolyticus* was subjected to N-terminal Edman sequencing by the NIAID Peptide Synthesis and Analysis Laboratory after removal of the N-terminal N-formyl modification by boiling in 25% TFA for 2 h at 55°C (Shively et al., 1982).

### Circular dichroism spectroscopy

The structure of synthetic PSM peptides was analyzed by CD spectroscopy on a Jasco spectropolarimeter model J-720. Solutions of PSM peptides, each at 1.0 mg/ml, were prepared in 50% trifluoroethanol. Measurements were performed in triplicate. Resulting scans were averaged, smoothed, and the buffer signal was subtracted.

### Measurement of hemolysis

Hemolysis (lysis of erythrocytes) was measured by incubating samples with human erythrocytes (2% in Dulbecco's phosphate-buffered saline, DPBS) for 1 h at 37°C as previously described (Wang et al., 2007). Hemolysis was determined by measuring OD_540nm_ using an ELISA reader.

### Measurement of neutrophil lysis

Lysis of human neutrophils by PSMs was determined essentially as described (Voyich et al., 2005). PSMs were incubated in 96-well tissue culture plates with 10^6^ neutrophils and plates were incubated at 37°C for 1 h. Then, neutrophil lysis was determined using a Cytotoxicity Detection Kit (Roche Applied Sciences) by release of lactate dehydrogenase (LDH).

### Measurement of neutrophil chemotaxis

Neutrophils were subjected to a brief hypotonic shock with pyrogen-free water, washed, and suspended at 5 × 10^6^ cells/ml in Hank's Balanced Salt Solution containing 0.05% human serum albumin. Chemotaxis of neutrophils was determined by using fluorescently-labeled neutrophils that migrated through a membrane fitted into an insert of a 24-well microtiter plate transwell system (Costar) containing a prewetted 3-μm-pore-size polycarbonate filter as described (de Haas et al., 2004).

### Statistical analysis

Statistical analysis was performed using Graph Pad Prism version 6.02. All error bars depict the standard deviation.

## Results

### Analysis of *S. haemolyticus* culture filtrate by RP-HPLC/MS

**Figure 1:**
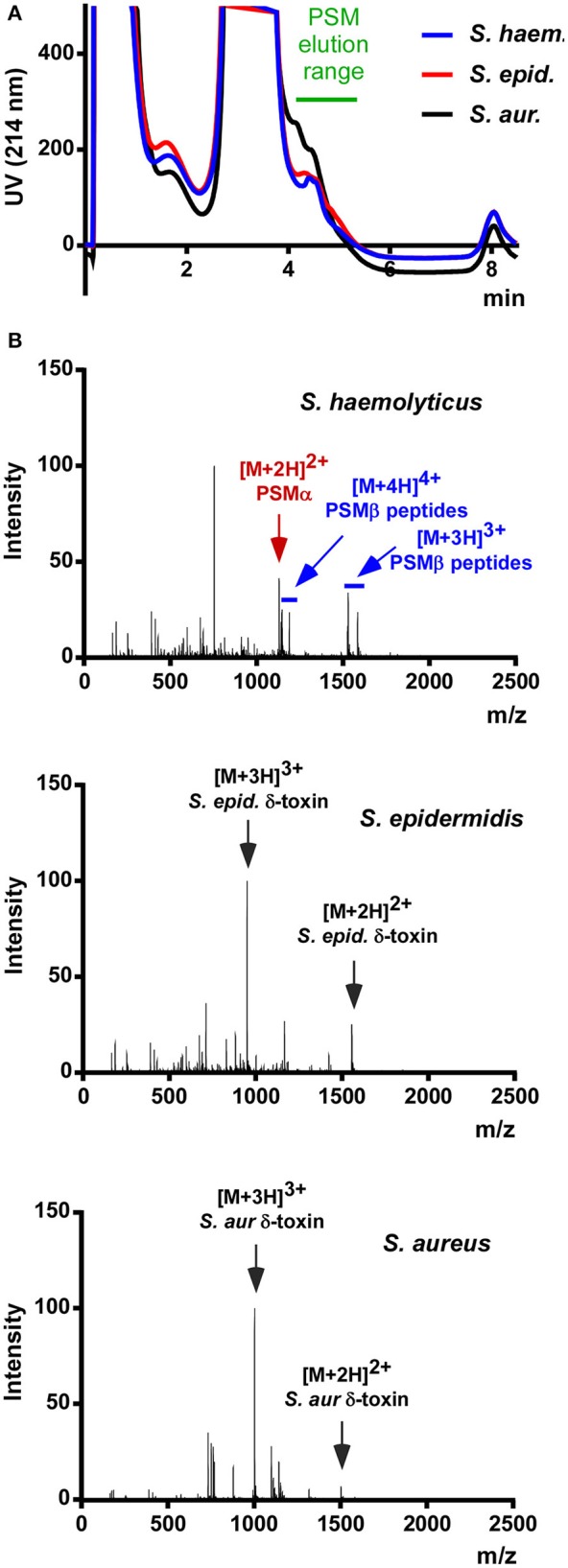
**RP-HPLC/MS of *S. haemolyticus* culture filtrate in comparison with *S. aureus* and *S. epidermidis***. **(A)** Stationary-phase culture filtrates of *S. haemolyticus, S. epidermidis*, and *S. aureus* were subjected to routine HPLC/MS detection of PSMs. **(B)** The averaged mass spectrum over that elution range showed the expected PSMs of *S. epidermidis* and *S. aureus*, among which the abundant m/z peaks belonging to δ-toxin are marked. *S. haemolyticus* showed a series of m/z peaks suggestive of three β-type PSMs and one α-type PSM.

As a preliminary test for the presence of PSMs in *S. haemolyticus*, we prepared stationary-phase (8 h) culture filtrate and subjected it to the rapid RP-HPLC/MS analysis of PSMs we routinely use in our laboratory. Stationary-phase cultures were used because PSM production has been shown to be strictly regulated by the accessory gene regulator (Agr) quorum-sensing system in *S. aureus* and *S. epidermidis* (Vuong et al., 2004a; Queck et al., 2008); and based on genome sequence analysis showing presence of Agr it is fair to assume that this is also the case in *S. haemolyticus* (Takeuchi et al., 2005). Analysis of culture filtrate showed that *S. haemolyticus* produces peaks in the elution range of PSMs with masses suggesting the presence of several PSMs (Figure 1). Notably, this analysis confirmed that *S. haemolyticus* in contrast to *S. aureus* and *S. epidermidis* does not produce a δ-toxin, as genome analysis had suggested (Takeuchi et al., 2005).

### Purification and identification of *S. haemolyticus* PSMs

**Figure 2:**
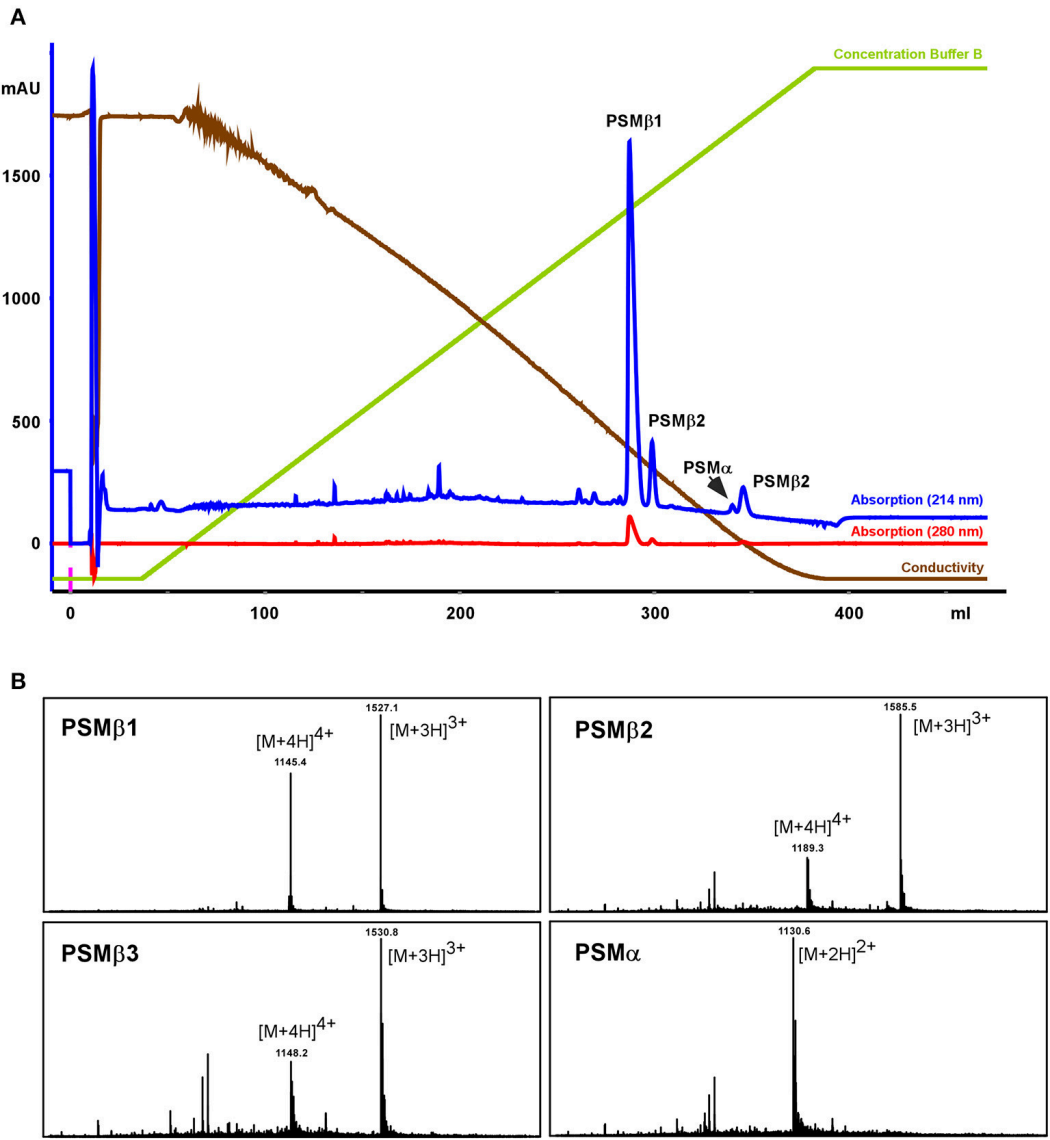
**Purification of *S. haemolyticus* PSMs**. *S. haemolyticus* PSMs were purified from stationary-phase culture filtrate by initial chromatography on SOURCE PHE material and subsequent high-resolution HPLC on C18 material, shown in **(A)**. Peaks in the elution range of PSMs were then further analyzed by RP-HPLC/MS **(B)**.

To further characterize those putative *S. haemolyticus* PSMs, a larger-scale purification was performed. Culture filtrate was subjected to initial RP chromatography on SOURCE PHE material and the fractions in the range where PSMs elute were lyophilized, resuspended, and subjected to high-resolution RP-HPLC on C18 material (see Section Methods for details; Figure 2). With this procedure, the four PSMs could be separated and isolated in sufficient amounts for clear mass spectrometric analysis using RP-HPLC/MS of the single fractions, as well as N-terminal sequencing.

All PSMs carry an N-terminal N-formyl modification at the N-terminal methionine, as present in all primary bacterial translation products, due to the lack of a signal peptide (Cheung et al., 2014a). In *S. aureus*, peptide N-deformylase removes this modification to a substantial degree, resulting in considerable amounts of N-deformylated PSMs in the culture filtrate (Cheung et al., 2010). In *S. haemolyticus*, similar to *S. epidermidis* (Czekaj et al., 2015), N-deformylation only occurred to a very minor degree, as evident from only small peaks with masses corresponding to N-deformylated PSMs.

### Amino acid sequences and genes of *S. haemolyticus* PSMs

**Figure 3:**
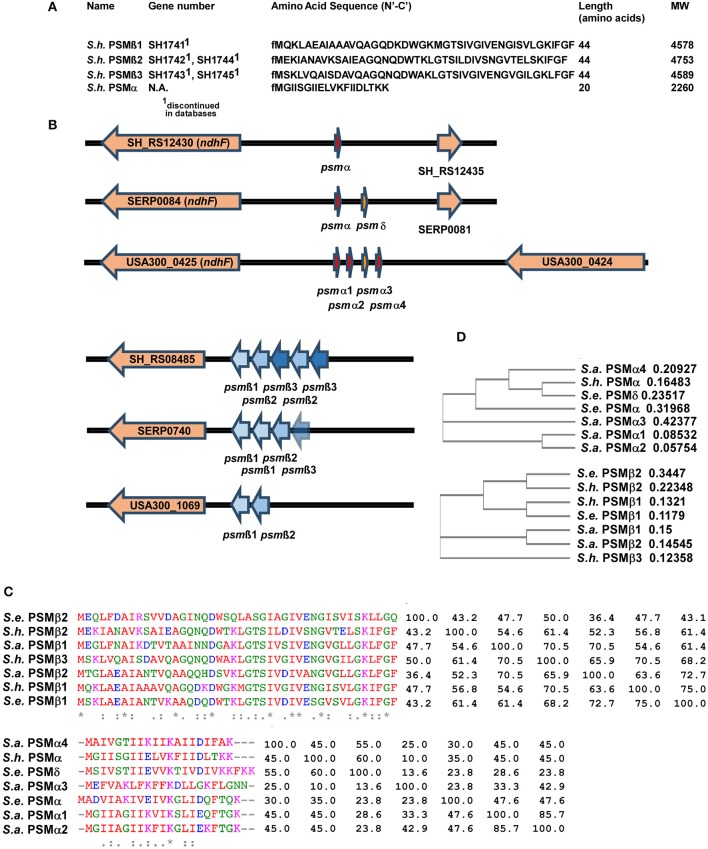
**Amino acid sequences and genes of *S. haemolyticus* PSMs and encoding loci in comparison with *S. aureus* and *S. epidermidis*. (A)** Amino acid sequences of *S. haemolyticus* PSMs determined by N-terminal sequencing and/or comparison of molecular weights with annotated open reading frames in the *S. haemolyticus* genome. **(B)** Location of PSM-encoding genes in the genome of *S. haemolyticus*, in comparison with corresponding loci in *S. aureus* and *S. epidermidis*. Gene numbers for *S. aureus* and *S. epidermidis* are those from the USA300 FPR3757 or RP62A strain genomes, respectively. **(C,D)** Alignments and calculated phylogenetic trees of PSMα and PSMβ peptides of *S. haemolyticus, S. aureus*, and *S. epidermidis*. Sequences were analyzed using the online tool Clustal Omega (http://www.ebi.ac.uk/Tools/msa/clustalo/). Next to the alignment in **(C)**, the pairwise similarity matrix is shown.

Masses obtained by HPLC/MS and in case of the α-type peptide, the entire sequence obtained by Edman sequencing, together with genome analysis, allowed clear identification of the amino acid sequences of the four PSMs of *S. haemolyticus* and the genetic loci in which they are encoded (Figures 3A,B). The three PSMβ peptides of *S. haemolyticus* (PSMβ1, PSMβ2, PSMβ3), which correspond in amino acid sequence to the previously described gonococcal growth inhibitor peptides, are encoded in an apparent operon in front of gene SH_RS08485. The genes encoding PSMβ2 and PSMβ3 are duplicated, a situation also known for some other PSM-encoding loci, notably the *S. epidermidis psm*β locus whose encoded PSMβ peptides share some similarity with the *S. haemolyticus* PSMβ peptides (Cheung et al., 2014a).

While the genes encoding the longer PSMβ peptides can often be found by computer similarity searches such as BLAST, on the peptide or DNA level, this is virtually impossible for the short α-type PSMs. Identification of the genetic locus encoding PSMα of *S. haemolyticus* thus depended on the amino acid sequence we obtained from Edman sequencing. This allowed attributing production of *S. haemolyticus* PSMα to a gene in the vicinity of the *ndhF* gene, a location, which also encodes the PSMα peptides of *S. aureus* and two α-type PSMs of *S. epidermidis* (Cheung et al., 2014a), suggesting a common origin (Figure 3B). It is noteworthy that an alignment with similarity analysis and phylogenetic analysis of the *S. aureus, S. epidermidis*, and *S. haemolyticus* PSMs showed that PSMs are sometimes very similar to other PSMs in the same locus, but sometimes more to PSMs in the corresponding locus in another species (Figures 3C,D). Thus, it appears that gene duplication events happened in evolution after, as well as before, the transfer of the *psm*α and *psm*β loci between species.

### *S. haemolyticus* PSMs form amphipathic α-helices

**Figure 4:**
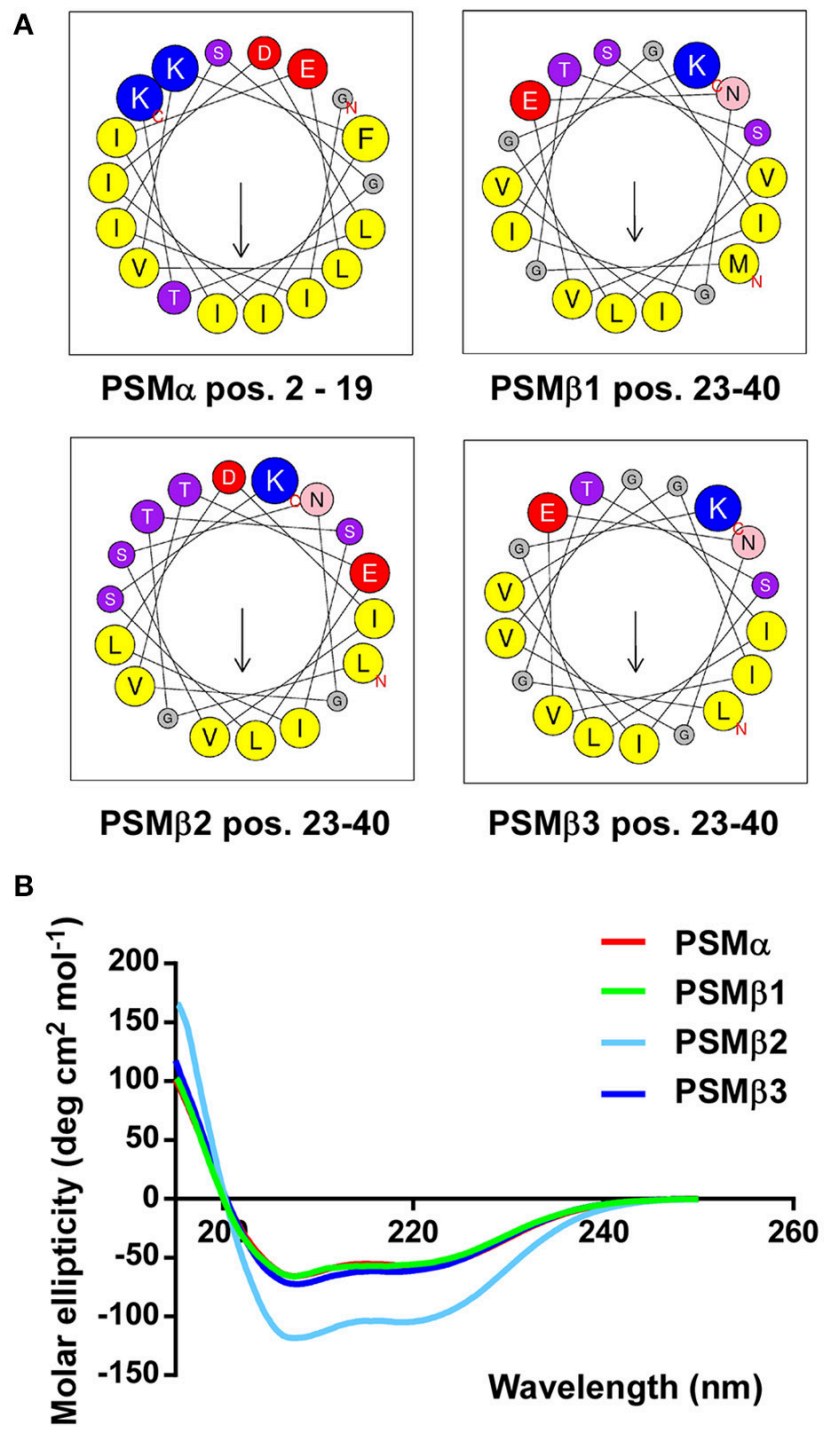
***S. haemolyticus*** **PSMs form amphipathic α-helices**. **(A)** Computation of α-helical wheels in the α-helical part of the peptides (using the program available at http://heliquest.ipmc.cnrs.fr). Note opposite arrangement of hydrophobic (yellow) vs. charged (red, blue) and hydroxyl (purple) amino acids. **(B)** Circular dichroism spectra of peptides in membrane environment conditions (50% trifluoroethanol).

Next, we computed α-helical wheels and measured α-helicity by circular dichroism (CD) analysis, to confirm that the *S. haemolyticus* PSMs also fulfill the structural requirements, i.e., formation of amphipathic α-helices, to be called PSMs. Indeed, all four *S. haemolyticus* PSMs are α-helical according to CD, as they showed the maximum at 193 nm and the two minima at 208 and 222 nm that are characteristic for α-helices (Figure 4A). Furthermore, when the sequences were arranged in α-helical wheels by bioinformatics analysis, they all showed opposite arrangement of hydrophilic and hydrophobic amino acids, characteristic of amphipathic α-helical peptides (Figure 4B).

### Production of *S. haemolyticus* PSMs is conserved among different isolates

**Figure 5:**
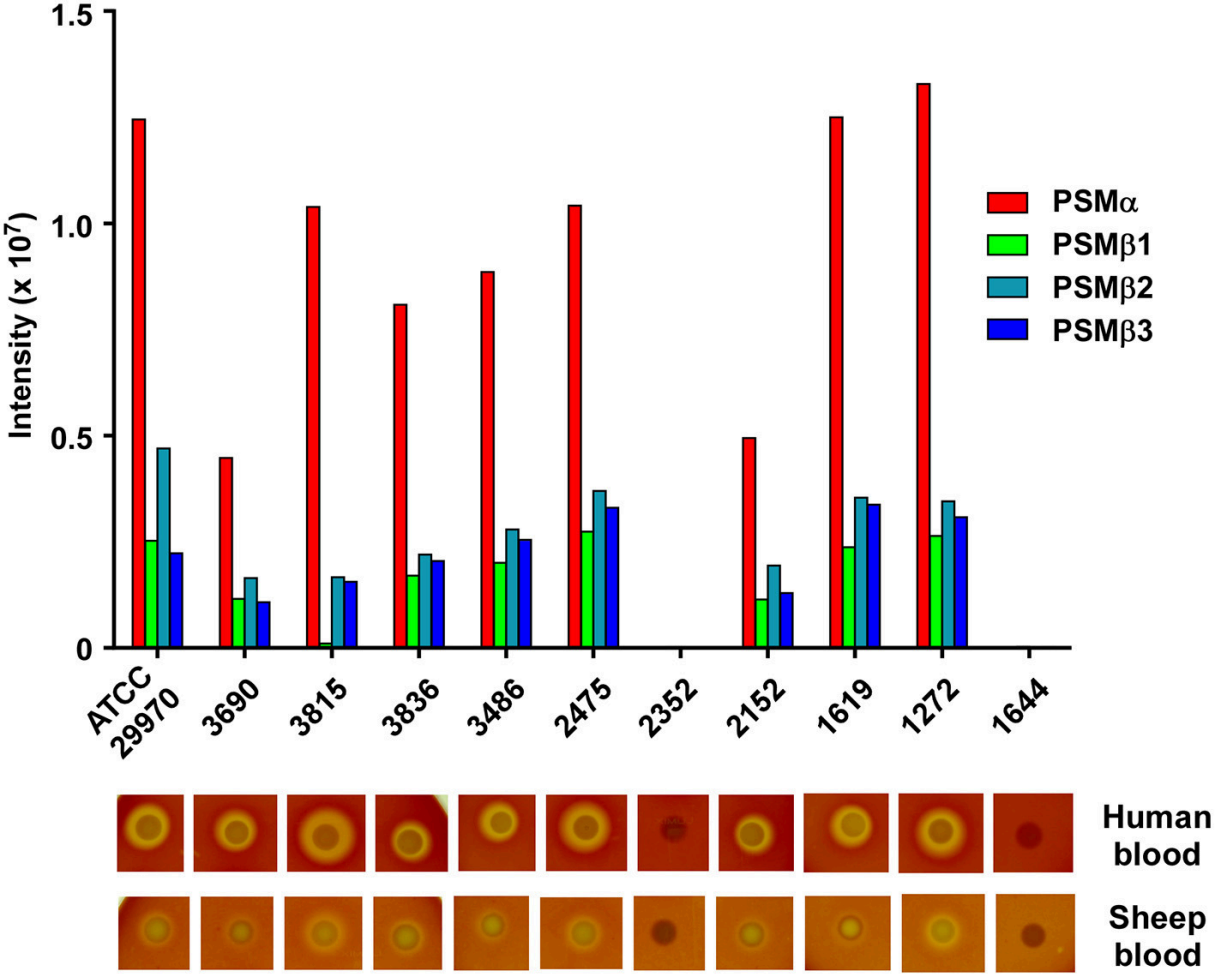
**Production of *S. haemolyticus* PSMs is conserved among different isolates and correlates with hemolytic capacity**. Stationary-phase culture filtrates of 11 *S. haemolyticus* isolates were analyzed by RP-HPLC/MS. Below, hemolytic capacity of isolates is shown. Isolates were analyzed by spotting on human or sheep agar plates and growing overnight in a 37°C incubator.

With the exception of the difference that is due to the presence of the mobile genetic element-encoded PSM-mec, based on evidence obtained with *S. aureus* and *S. epidermidis*, the PSM pattern of a given staphylococcal species is conserved among different isolates and strains (Wang et al., 2007; Queck et al., 2009). Therefore, we analyzed whether the PSM pattern is also conserved in different *S. haemolyticus* strains. To that end, we analyzed 10 *S. haemolyticus* bacteremia blood culture isolates obtained from the Eppendorf University Hospital in Hamburg, Germany, in addition to the ATCC29970 strain used for analysis and purification. Eight of the ten strains showed the same PSM pattern in terms of presence of the four PSM peptides, with only slight differences in production (Figure 5). In one of the eight strains, no PSMβ1 production was observed, with the remaining pattern being conserved, suggesting a possible point mutation or deletion abolishing production of that peptide. Two strains were devoid of PSM production, a situation with similar frequency also seen in *S. aureus* and *S. epidermidis* (Vuong et al., 2004b; Traber et al., 2008), originating from spontaneous mutations in the Agr system, which strictly controls PSM production (Queck et al., 2008). Notably, when tested on human or sheep blood, the PSM-deficient strains lacked hemolytic capacities, while PSM producers were all hemolytic, indicating that PSM production underlies hemolytic activity in *S. haemolyticus*. Interestingly, hemolytic activity was in general more pronounced toward human than sheep blood in this assay.

### Hemolytic activities of *S. haemolyticus* PSMs

**Figure 6:**
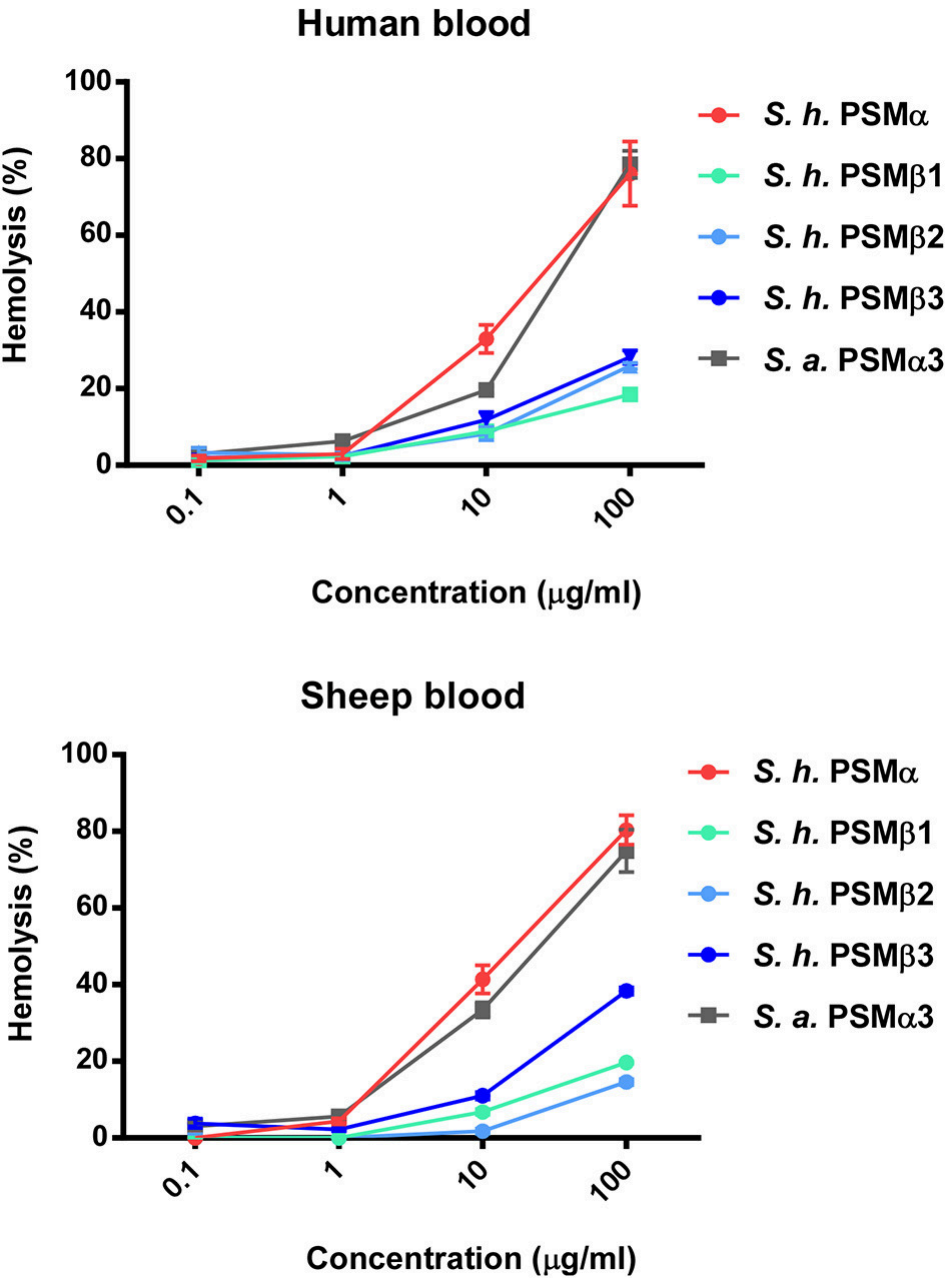
**Hemolytic activities of *S. haemolyticus* PSMs**. Hemolytic activities of *S. haemolyticus* PSMs at different concentrations were measured using incubation with human erythrocytes and compared to that achieved with equal amounts of PSMα3 of *S. aureus*. Activities are compared as percentage compared to a control with total lysis. Data are from assays performed in quadruplicate.

Hemolytic activity is what gave *S. haemolyticus* its name; however, hemolytic agents were never clearly identified in *S. haemolyticus*, although it was suspected that they may be peptides (Loyer et al., 1990). To investigate whether the identified PSMs of *S. haemolyticus* have hemolytic activity that could explain the hemolytic properties of that species, we measured lysis of human erythrocytes. All *S. haemolyticus* PSMs showed hemolytic capacity, with that of the PSMα peptide in particular reaching levels known for the most hemolytic peptides in other staphylococci, PSMα3 of *S. aureus* and PSMδ of *S. epidermidis* (Wang et al., 2007; Cheung et al., 2010), as demonstrated by direct comparison with *S. aureus* PSMα3 (Figure 6). This peptide is thus a good candidate to account for the hemolytic capacity of *S. haemolyticus*; however, also the PSMβ peptides may have a significant contribution to the hemolytic capacity of *S. haemolyticus* given that they are produced at high levels (see Figure 2). Hemolytic capacity was similar in this assay toward human vs. sheep blood.

### Cytolytic activities of *S. haemolyticus* PSMs toward human neutrophils

**Figure 7:**
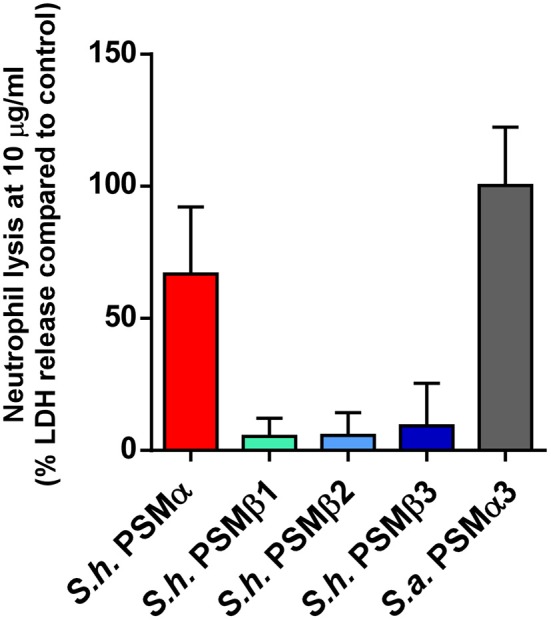
**Cytolytic activities of *S. haemolyticus* PSMs toward human neutrophils**. Cytolytic activities of *S. haemolyticus* PSMs and *S. aureus* PSMα3 at 10 μg/ml were measured using incubation with human neutrophils by release of lactate dehydrogenase (LDH). Activities are compared as percentage compared to a control with total lysis. Triplicate measurements were performed each using neutrophils obtained from three donors.

Phagocytosis and elimination by neutrophils is the preeminent way host defense deals with invading staphylococci (Rigby and DeLeo, 2012). The *psm*α locus of *S. aureus* has been shown to be crucial for the elimination of neutrophils and thus to represent a key part of the immune evasion capacity of *S. aureus* (Wang et al., 2007). While *S. epidermidis* PSMs other than PSM-mec have not yet been investigated for their contribution to that phenotype using isogenic deletion mutants, cytolytic activity toward human neutrophils is found in particular for *S. epidermidis* PSMδ (Cheung et al., 2010). In contrast to α-type PSMs, β-type PSMs are commonly found to have only very low cytolytic activity toward leukocytes (Wang et al., 2007; Cheung et al., 2010). Thus, we measured lysis of human neutrophils by release of lactate dehydrogenase for all *S. haemolyticus* PSMs. As expected, we detected only very low cytolytic activity toward human neutrophils by *S. haemolyticus* PSMβ peptides. However, the cytolytic activity of *S. haemolyticus* PSMα was very high, reaching activity levels exerted by the strongly lytic *S. aureus* PSMα3 (Figure 7).

### Propensities of *S. haemolyticus* PSMs to elicit chemotaxis by human neutrophils

**Figure 8:**
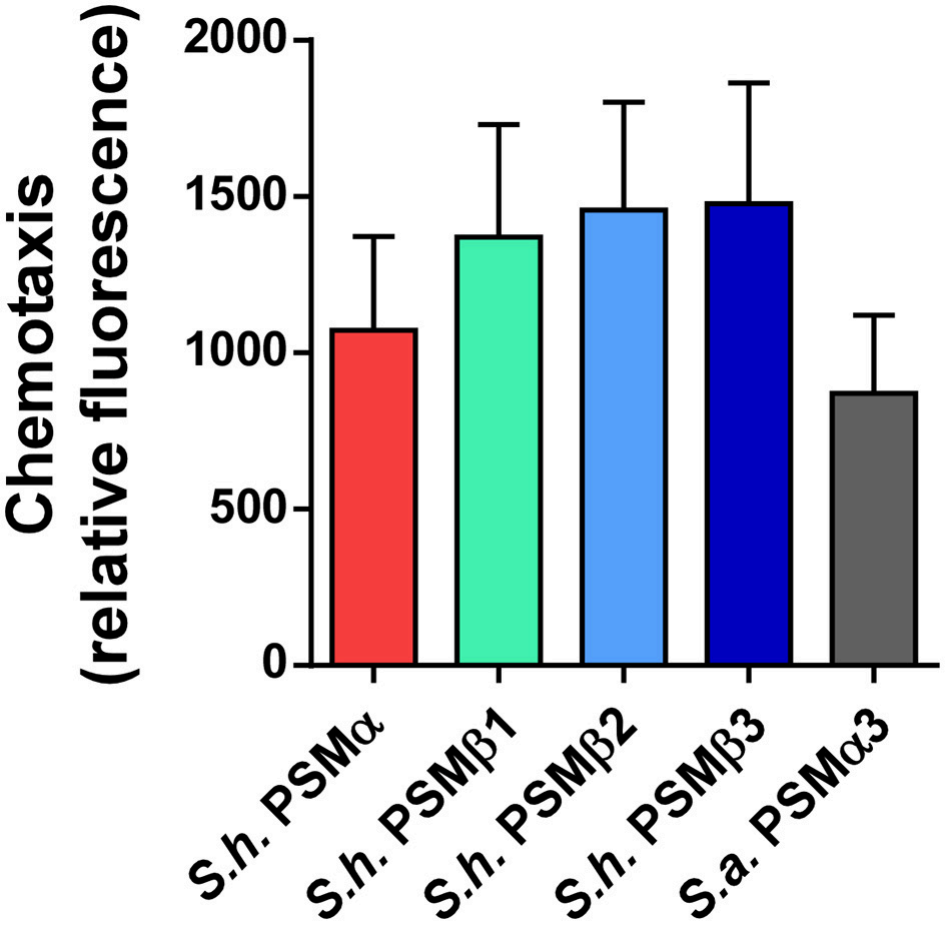
**Chemotactic activities of *S. haemolyticus* PSMs toward human neutrophils**. Chemotactic activities of *S. haemolyticus* PSMs and *S. aureus* PSMα3 toward human neutrophils were determined using a transwell system. PSMs were applied at a concentration of 5 mM.

All PSMs described to date bind to FPR2 and activate neutrophils (Kretschmer et al., 2010). This is likely to be interpreted as a way of the human immune system to recognize staphylococcal invaders. Arguably the most crucial phenotype that is triggered by that interaction is neutrophil chemotaxis, i.e., the attraction of neutrophils to the site of staphylococcal infection. Although, PSM-FPR2 interaction is poorly understood, different structural features than those that promote receptor-independent cytolysis likely determine the receptor-dependent pro-inflammatory activities (Cheung et al., 2014b). Thus, it is not uncommon to find similar pro-inflammatory capacities in PSMs that differ very much in their cytolytic capacities. Indeed, when we measured chemotaxis as an important inflammatory phenotype that is triggered by PSMs in human neutrophils, we found that all *S. haemolyticus* PSMs were similarly pro-inflammatory (Figure 8).

## Discussion

The term “PSMs” describes a recently discovered family of pro-inflammatory and cytolytic staphylococcal peptide toxins (Cheung et al., 2014a). In *S. aureus*, it has been shown that PSMs contribute significantly to several disease manifestations (Wang et al., 2007). CoNS also frequently cause infections, such as particularly blood infections, but they are not generally known to produce toxins (Becker et al., 2014). Thus, members of the PSM family are premier candidates to explain those yet poorly understood virulence characteristics of CoNS. However, a complete investigation of the nature, production levels, cytolytic, and pro-inflammatory capacities of PSM peptides has only been performed in the CoNS species *S. epidermidis* (Cheung et al., 2010). Notably, together with preliminary characterization of other CoNS species by mass spectrometry (Rautenberg et al., 2011), these analyses also showed that PSMs vary considerably in pattern, sequence, and production levels between species, making a detailed specific investigation necessary for each species in question. Furthermore, due to the short length of the open reading frames encoding in particular the short α-type peptides, mere genome searches can hardly be used to identify which PSMs are encoded in a given strain or species.

*S. haemolyticus* is a virulent CoNS species and, after *S. epidermidis*, the species that is second in terms of causing CoNS infections. It is particularly notorious for blood infections, with its name being reminiscent of the involvement with this infection type (Czekaj et al., 2015). However, the molecular basis of hemolysis in *S. haemolyticus* has remained unknown. Therefore, in the present study, we systematically analyzed an *S. haemolyticus* standard strain for the presence of PSMs as putative hemolysins and detected three PSMβ peptides, which are identical to the previously described gonococcal growth inhibitor peptides of *S. haemolyticus* (Watson et al., 1988). Notably, we also identified a previously not described α-type PSM, which we named PSMα of *S. haemolyticus*. Furthermore, we confirmed on the protein level what genome analysis had suggested concerning the production of δ-toxin in *S. haemolyticus*, namely, that this species, in contrast to many other staphylococci, does not produce a δ-toxin. Analysis of a series of *S. haemolyticus* clinical isolates showed that this PSM pattern is conserved within the species, confirming the notion, based on the analysis of *S. epidermidis* and *S. aureus* isolates, that the PSM pattern is characteristic for a staphylococcal species. *S. haemolyticus* represents only the third staphylococcal species for which the PSM pattern has been systematically analyzed. Whether the PSM pattern can be used generally for staphylococcal species identification will need to be shown using more species in the future.

Our study shows that especially the novel PSMα peptide of *S. haemolyticus*, but also to some degree the PSMβ peptides, have hemolytic activity, strongly suggesting—in particular in the absence of other known hemolysins in *S. haemolyticus* (Takeuchi et al., 2005; Czekaj et al., 2015)—that these peptides are the key hemolytic agents of the species. Similar to other strongly hemolytic PSMs, such as PSMα3 of *S. aureus* (Wang et al., 2007), the hemolytic capacity was paired with pronounced cytolytic capacity toward human neutrophils, indicating a role of particularly PSMα in the immune evasion properties of *S. haemolyticus*. It is especially intriguing, and emphasizing the need to appreciate the unique nature of every identified PSM, that the amino acid similarity between the two equally and strongly hemolytic α-type PSM peptides of, *S. aureus* PSMα3 and *S. haemolyticus* PSMα, respectively, is only 10%, a value that would not indicate a common origin or relatedness in online similarity searches.

In contrast to the cytolytic properties of PSMs, their pro-inflammatory activity is mostly due to direct or indirect activation of pathogen recognition receptors (PRRs). Underlining that difference, all identified *S. haemolyticus* PSMs had considerable pro-inflammatory capacity, as indicated by their chemotactic potencies toward human neutrophils, in contrast to their strongly varying cytolytic capacities. This is of particular importance given that one of the hallmarks of sepsis is an overwhelming immune response to infection. However, the pathogenesis of sepsis is poorly understood and it remains to be seen for the specific case of CoNS-mediated sepsis, whether PSMs contribute to the development of sepsis by eliminating immune cells via their cytolytic capacities or by causing an immune overreaction with cytokine storm via their pro-inflammatory, receptor-dependent capacities. In that regard, it is also worth noting that N-deformylation, similar to *S. epidermidis* but in contrast to *S. aureus* (Cheung et al., 2010), did not occur in *S. haemolyticus* to a considerable extent. The removal of N-formyl methionine by peptide N-deformylase is considered a virulence factor, as N-formylated peptides are pathogen-associated molecular patterns. PSMs compose a considerable part of the total secreted proteins, as shown in *S. aureus* (Chatterjee et al., 2013), and presumably even to a higher relative degree in many CoNS, suggesting that the degree of PSM N-deformylation matters for immune evasion. Then again, also N-deformylated PSMs activate the formyl peptide receptor 2, the main receptor directly recognizing PSMs, to a significant degree. Thus, it is difficult to dissect and remains to be shown, whether the degree of N-formylation in PSMs plays a significant role for host-pathogen interaction.

Substantiating the idea that PSMs are mainly responsible for the hemolytic properties of *S. haemolyticus*, there was a clear correlation between PSM production and hemolytic capacity in a collection of clinical *S. haemolyticus* strains. PSM-deficient isolates, most likely mutants in the PSM regulator Agr, were devoid of hemolytic capacity on sheep or human blood. However, we are aware that to unambiguously show that hemolysis in *S. haemolyticus* is entirely due to PSMs, the analysis of isogenic mutants will be required. The fact that isogenic gene deletion mutants are extremely difficult to prepare in CoNS so far has prevented us from analyzing the contribution of PSMs to infection and immune evasion *in vitro* and *in vivo* in their natural strain background. This is even more challenging given the multiplicity of PSMs and their functional redundancy, making it necessary to produce sequential, multiple *psm* gene locus knockouts. Notably, no isogenic gene deletion mutants have yet been produced in *S. haemolyticus* and it is unclear whether this species is amenable to genetic engineering at all. However, we are aware of the necessity of producing and analyzing such *psm* gene deletion mutants and are currently working toward this goal. These mutants will also be helpful in analyzing the contribution of PSMs to further phenotypes, such as biofilm development, which are difficult to analyze using peptides alone. Finally, we are working toward producing an *agr* deletion mutant in *S. haemolyticus* to confirm that PSMs in that species are also under control of that quorum-sensing virulence regulator, similar to *S. aureus* and *S. epidermidis*.

In conclusion, this is the first study that characterizes toxins of *S. haemolyticus*. The fact that they have strong cytolytic activities toward human erythrocytes and leukocytes indicates a key role as virulence factors in that species.

## Author contributions

FD, HJ, GC, and AV performed experiments. FD, HJ, GC, and MO analyzed data. HR collected and provided *S. haemolyticus* blood isolates. MO and XL designed the study. MO wrote the paper.

### Conflict of interest statement

The authors declare that the research was conducted in the absence of any commercial or financial relationships that could be construed as a potential conflict of interest.
